# Evaluation of the Efficacy of Two New Biotechnological-Based Freeze-Dried Fertilizers for Sustainable Fe Deficiency Correction of Soybean Plants Grown in Calcareous Soils

**DOI:** 10.3389/fpls.2019.01335

**Published:** 2019-11-08

**Authors:** Carlos M. H. Ferreira, Sandra López-Rayo, Juan J. Lucena, Eduardo V. Soares, Helena M. V. M. Soares

**Affiliations:** ^1^REQUIMTE/LAQV, Departamento de Engenharia Química, Faculdade de Engenharia, Universidade do Porto, Porto, Portugal; ^2^Bioengineering Laboratory-CIETI, Chemical Engineering Department, ISEP-School of Engineering of Polytechnic Institute of Porto, Porto, Portugal; ^3^CEB-Centre of Biological Engineering, University of Minho, Braga, Portugal; ^4^Departamento de Química Agrícola y Bromatología, Facultad de Ciencias, Universidad Autónoma de Madrid, Madrid, Spain

**Keywords:** biotechnological-based iron fertilizers, sustainable agriculture, freeze-dried iron-bacterial siderophores products, environmental-friendly iron-chelates, iron chlorosis correction of soybean plants

## Abstract

Currently, fertilization with synthetic chelates is the most effective agricultural practice to prevent iron (Fe) deficiencies in crops, especially in calcareous soils. Because these compounds are not biodegradable, they are persistent in the environment, and so, there is the risk of metal leaching from the soils. Thus, new, more environment-friendly efficient solutions are needed to solve iron-deficiency-induced chlorosis (IDIC) in crops grown in calcareous soils. Therefore, the central aim of this work was to prepare new freeze-dried Fe products, using a biotechnological-based process, from two siderophores bacterial (*Azotobacter vinelandii* and *Bacillus subtilis*) cultures (which previously evidenced high Fe complexation ability at pH 9) and test their capacity for amending IDIC of soybean grown in calcareous soils. Results have shown that *A. vinelandii* iron fertilizer was more stable and interacted less with calcareous soils and its components than *B. subtilis* one. This behavior was noticeable in pot experiments where chlorotic soybean plants were treated with both fertilizer products. Plants treated with *A. vinelandii* fertilizer responded more significantly than those treated with *B. subtilis* one, when evaluated by their growth (20% more dry mass than negative control) and chlorophyll development (30% higher chlorophyll index than negative control) and in most parameters similar to the positive control, ethylenediamine-di(*o*-hydroxyphenylacetic acid). On average, Fe content was also higher in *A. vinelandii*-treated plants than on *B. subtilis*-treated ones. Results suggest that this new siderophore-based formulation product, prepared from *A. vinelandii* culture, can be regarded as a possible viable alternative for replacing the current nongreen Fe-chelating fertilizers and may envisage a sustainable and environment-friendly mending IDIC of soybean plants grown in calcareous soils.

## Introduction

Iron (Fe) deficiency-induced chlorosis (IDIC) is a yield-limiting factor in an extensive range of crops worldwide (Hansen et al., 2006; Briat et al., 2015), especially severe in calcareous soils. It is estimated that calcareous soils cover ∼30% of the world’s land surface (Hansen et al., 2006). In the specific case of soybean culture, the incidence of Fe deficiency is increasing and potentially affects nearly two million hectares (Hansen et al., 2006). Therefore, new, cheaper, and more environment-friendly iron fertilizer strategies are needed to overcome this agronomic problem.

Although commonly found in soil, due to its low solubility and dissolution kinetics, Fe availability for plants is very limited, especially in aerated alkaline and calcareous soils (Mengel, 1994; Shenker and Chen, 2005). Fe is essential for, but not only, chlorophyll synthesis; as a result, insufficient Fe in leaves causes low chlorophyll levels and the resultant yellowing of new leaves (Lucena, 2000; Chatterjee et al., 2017). As a consequence, many crop yields are negatively affected and impaired by low Fe bioavailability in soils (Martins et al., 2017).

The strategic requirements for the production of sustainable crops should: i) have a low impact on the environment, ii) be feasible, and iii) have low costs. However, in the case of the Fe fertilizers, available nowadays, these objectives are far from being reached. The most effective Fe correctors used in neutral and alkaline soils [mostly aminopolycarboxylic acid (APCAs) compounds, such as *o,o*-ethylenediamine-di(*o*-hydroxyphenylacetate)-Fe(III) and *o*,*o*-EDDHA/Fe(III) chelate (García-Marco et al., 2006; Bin et al., 2016)] have scarce degradability (Bucheli-Witschel and Egli, 2001) and can only be applied in cash crops due to their high price (Rajaie and Tavakoly, 2018). Thus, the introduction of alternative cheaper compounds with Fe-complex forming comparable to those synthetic compounds and showing better biodegradability is needed.

Many siderophores (namely those containing catechol and hydroxamic acid groups), which are produced by microorganisms in response to low Fe availability in the environment, are Fe-effective chelators, more selective to Fe(III) than to divalent metals and better biodegradable than synthetic APCAs (Pierwola et al., 2004; Crumbliss and Harrington, 2009; Fazary et al., 2016). These facts, together with others that demonstrated that microbial cultures containing siderophores were able to supply Fe to plants when cultivated in hydroponic medium (Yehuda et al., 1996; Chen et al., 2000; Hördt et al., 2000), suggest that siderophores may have potential to substitute the synthetic APCAs traditionally used for Fe fertilization in calcareous soils. However, the direct application of microbial cultures containing siderophores on soils is not feasible and raises several problems. The handling (transport and storage) and direct use of the supernatants of the microbial culture containing siderophore(s) for the fertilization of soils is not a feasible solution due to the large volumes needed. Moreover, siderophores are natural compounds and, thus, potentially biodegradable. A long storage period of the supernatants of these cultures would undoubtedly lead to partial or complete degradation of the siderophores with the consequent decrease of its concentration and effectiveness. Therefore, in this work, our first aim was to prepare sustainable Fe-chelate fertilizers based on a novel formulation made of freeze-dried products of two filtrates of cultures of *Azotobacter vinelandii* and *Bacillus subtilis*.

The formulation of a Fe fertilizer for Fe chlorosis correction of plants grown in calcareous soils constitutes a much vaster and complex problem. The performance of a Fe chelate to supply Fe to plants depends on numerous interactions of Fe chelates with soils that may reduce their presence in the soil solution. Among them, replacement of Fe from the chelates by other metal ions, namely, Ca^2+^ and Mg^2+^, which are present at high concentrations in calcareous soils and/or sorption of the Fe chelate(s) onto the soil surfaces regulate the Fe-chelate concentration in solution. Thus, to evaluate the ability of the new freeze-dried products for maintaining Fe in solution, their interactions with soil components and soil were initially assessed.

As it was mentioned above, Fe deficiency continues to be a challenge in soybean production. Therefore, the efficiency of the two freeze-dried formulations in mending IDIC in soybean plants grown in calcareous soil was studied by measuring plant development (dry mass), chlorophyll production, and plant tissue Fe content.

At the end, it is expected that this innovative strategy will contribute to producing environment-friendly (chelating compounds of biological origin constitute it) and cheaper (no separation processes of siderophores, which increase the price of the final product, were employed) Fe-chelate fertilizers that overcome the problems associated with the handling, transport, and storage of microbial cultures and are effective for correcting iron deficiencies of soybean plants grown in calcareous soil conditions.

## Materials and Methods

### Preparation of the Freeze-Dried Products

In this study, two bacteria strains were used: *A. vinelandii* Deutsche Sammlung von Mikroorganismen und Zellkulturen (DSM) 2289 and *B. subtilis* DSM 10. A. *vinelandii* was grown in Burk’s medium (BM) (Newton et al., 1953), whereas *B. subtilis* was grown in a minimal mineral medium (MM).

Bacterial filtrates were produced as described elsewhere (Ferreira et al., 2019b). Shortly, bacteria were grown in Fe-depleted media until siderophore concentration was stable (72 and 48 h for *A. vinelandii* and *B subtilis*, respectively). Bacterial biomass was pelleted by centrifugation, and the supernatant was filtered through a 0.45-µm pore size filter. The resulting filtrate was mixed with corn starch (5 and 15 g L^−1^ for *B. subtilis* and *A. vinelandii*, respectively), which was used as an anticaking agent. Then, the mixture was freeze dried (Labconco FreeZone 2.5 L coupled with a VacuuBrand RC 6 pump, USA). The resulting powder was homogenized and stored at 4°C until use.

### Preparations of Iron Siderophore Solutions

#### Solution and Soil Interaction Assays

To study the suitability of each bacterial media for Fe chlorosis remediation in soil applications, an Fe-siderophore solution (ISS) was prepared by rehydrating to the same volume the previously obtained powders; this procedure allowed to achieve the same initial concentration of siderophores. Then, a soluble salt of Fe(III), FeCl_3_.6H_2_O (Sigma), was added to the maximum complexation capacity at pH 9.0 (*A. vinelandii* ≈ 188 µmol L^−1^ and *B. subtilis* ≈ 225 µmol L^−1^) of each solution, which was previously determined (Ferreira et al., 2019b). During the process, the pH of the solution was kept between 7.0 and 9.0, and the final pH was set at 9.0. The solution was allowed to settle overnight and filtered (0.45-µm pore size filter). The initial Fe concentration was then measured by atomic absorption spectroscopy with flame atomization (AAS-FA) using a Perkin-Elmer AAnalyst 400 spectrometer (Norwalk, CT, USA).

#### Application in Calcareous Soil and Soybean Response

For soybean chlorosis amendment, a more concentrated ISS was used. Therefore, ISS were produced by dissolving the powder, obtained previously, in deionized water (20 and 120 g L^−1^ for *B. subtilis* and *A. vinelandii*, respectively) and a soluble salt of Fe(III), FeCl_3_.6H_2_O, was added to achieve 120 and 110 mg L^−1^ of Fe for *B. subtilis* and *A. vinelandii*, respectively. In the process, the pH of the solution was kept between 7.0 and 9.0, and the final pH was set at 9.0. The solutions were allowed to settle overnight and filtered (0.45-µm pore size filter). The initial Fe concentration was then measured similarly, as described in Section 2.2.1.

### Equilibrium and Stability of Fe Chelates in Soil

To understand the suitability of each ISS for Fe chlorosis amendment of soybean plants in soil applications, a sequence of tests was performed.

The first test addressed the effect of pH solution and the presence of Ca^2+^ on the ISS stability. Five sets of replicas (three per set), corresponding to the pH values tested, were prepared by mixing 5 ml of ISS with 5 ml of a buffered solution [10 mmol L^−1^ 4-(2-hydroxyethyl)-1-piperazineethanesulfonic acid, 10 mmol L^−1^ 2-(*N*-morpholino)ethanesulfonic acid, 10 mmol L^−1^ *N*-cyclohexyl-3-aminopropanesulfonic acid, and 10 mmol L^−1^ *N*-cyclohexyl-2-hydroxyl-3-aminopropanesulfonic acid] of 100 mmol L^−1^ CaCl_2_. The tubes were covered with aluminum foil to prevent light exposure. Then, the pH of each set of replicas was adjusted to 7.0, 7.5, 8.0, 8.5, and 9.0, respectively. The tubes were shaken (150 revolutions per min, rpm) in an orbital shaker (Grant Ols200, UK) at 25°C for 3 days. In the end, samples were filtered (0.45-µm pore size filter), acidified with nitric acid (HNO_3_) (Merck) (1%), and the soluble Fe was determined as described above.

The second test consisted of the interaction study of ISS with different soils and soil components. For this purpose, 25 mL of each ISS was added separately to two types of soil (5.0 g): a sandy clay soil from Picassent (Valencia, Spain) and a standard soil prepared in our lab, as described by Álvarez-Fernández et al. (1997); individual soil constituents were also tested: CaCO_3_ (2.0 g), Ca-montmorillonite (0.5 g), goethite (0.25 g), ferrihydrite (0.25 g), and organic matter (0.5 g). Tubes were covered with aluminum foil to prevent light exposure, and each experimental condition was conducted in triplicate. Samples were shaken (150 rpm, 1 h) in an orbital shaker (Grant Ols200, UK) and then left to stand for 3 days at 25°C. In the end, samples were filtered (0.45-µm pore size filter), acidified with HNO_3_ (1%), and the soluble Fe was determined by AAS-FA, as described above.

The third and last test was conducted using a mixture of the soils with calcareous sand (50/50%), for a total of 5 g. To the mixture, 0.5 mL of ISS was added, intercalating with 0.5 mL of deionized water, before and after (for a total of 1.5 mL added). This resulted in a more reactive condition as the soil/solution ratio was higher [soil/solution (g mL^-1^) = 3.33] compared to the one used in the previous experiment [soil/solution (g mL^-1^) = 0.20]. Samples were kept in the dark at 25°C for 3 days, and the moisture level of the soils was checked regularly and maintained by adding water when necessary. Three replicates were done for each ISS. At the end of the 3 days, the soluble Fe was extracted by adding 10 mL of water to each sample, which was then manually shaken and filtered. Finally, the soluble Fe was determined by AAS-FA, as described above.

### Evaluation of the Soybean Response to Fe Chelates Application in Calcareous Soil

#### Experimental Setup

First, *Glycine max* cv. AG 1833 seeds were germinated in perlite on sterile trays placed in distilled water. Trays were kept at 30°C and 60% relative humidity for 4 days, and the water level inside the trays was monitored to prevent dryness.

Seedlings were then examined, selected, and transferred to a tray, with a perforated plate floating on top of nutrient solution [macronutrients (mmol L^−1^): 1.0 Ca(NO_3_)_2_, 0.9 KNO_3_, 0.3 MgSO_4_, and 0.1 KH_2_PO_4_; buffered micronutrients (μmol L^–1^): 2.5 MnSO_4_, 1.0 CuSO_4_,10 ZnSO_4_, 1.0 NiCl_2_, 1.0 CoSO_4_, and 115.5 Na_2_EDTA; other micronutrients (μmol L^−1^): 35 NaCl, 10 H_3_BO_3_, and 0.05 Na_2_MoO_4_. The pH was buffered with 0.1 mmol L^−1^ 4-(2-hydroxyethyl)-1-piperazineethanesulfonic acid and adjusted to 7.5 with 1.0 mol L^−1^ KOH] (Nadal et al., 2012) and left for 7 days. Then, plants were transferred to the experiment pots.

Methacrylate cylinders (7 cm diameter, 16 cm high) were used as pots, coated with aluminum foil on the outside to protect the content from light and a piece of mesh on the bottom to allow aeration. A mixture of sandy clay soil (70%) from Picassent (Valencia, Spain) and calcareous sand (30%), in a total of 0.6 kg, was used to fill the pots. Two days before the plants’ transfer, pots were irrigated to 80% of the soil water capacity and, henceforth, watered with sufficient solution to preserve it. The irrigation was conducted using a pH buffered nutrient solution containing the same macronutrients and 1 mmol L^−1^ of CaCO_3_ and 1.19 mmol L^−1^ of NaHCO_3_ (pH 8.0–8.5). The pots were placed on Petri dishes plates to control chelate leaching. The experiment was carried out in a growth chamber (Dycometal-type CCKF 0/16985) using a daily cycle of 16 h/8 h (day/night); at day: 25°C and a relative humidity of 40%; at night: 20°C with a relative humidity of 60%.

**Figure 1:**
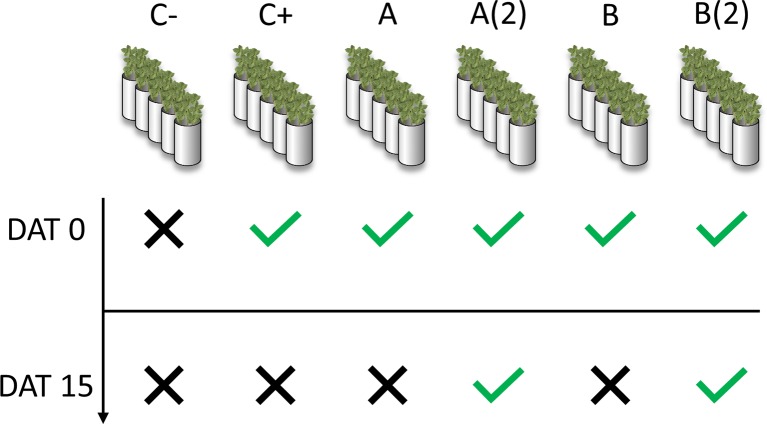
Treatment schedule for application of iron siderophore solutions (ISS) to chlorotic soybean in calcareous soils. DAT: days after treatment; C−: negative control; C+: positive control (o,o-EDDHA); **(A)** *A. vinelandii* ISS; **(B)** *B. subtilis* ISS. **A(2)** and **B(2)** correspond to second application of *A. vinelandii* ISS and *B. subtilis* ISS, respectively.

A total of six-pot sets were prepared: one without any treatment (negative control, C−), one for *o,o*-EDDHA (positive control, C+), two for *A. vinelandii* ISS (A), and two for *B. subtilis* ISS (B). Each pot set had five pots, totaling five pots for the negative control, five for positive control, and ten for *A. vinelandii* [A and A(2)] and *B. subtilis* ISS [B and B(2)], respectively, according to the scheme presented in **Figure 1**. To each pot (unless negative control pots), enough volume of chelate or ISS solution was applied to have 2.5 mg of Fe(III) per kilogram of soil. At the 15th day after the first treatment applications, to one set (five pots) of each *A. vinelandii* and *B. subtilis* sets [A(2) and B(2), respectively], a second treatment was applied (**Figure 1**). Plants were harvested 7 and 21 days after treatment (DAT) application. Major nutrients (K^+^, Mg^2+^, PO_4_^3-^, NO_3_^−^, NH_4_^+^, and Ca^2+^) present in ISS were quantified and balanced by the addition of appropriate nutrient solutions (**Table S1**) so that the same quantity of major nutrients was present in all pot sets. Solutions A and C were used for the correction of Ca^2+^ and NO_3_^−^, and Mg^2+^, respectively. As *A. vinelandii* ISS and *B. subtilis* ISS had different K^+^ and PO_4_^3−^ concentrations, solution B was used to equal these nutrients between the two treatments, while solution D was used to balance the K^+^ and PO_4_^3−^ content on the soils where no ISS treatment was applied.

#### Plant and Soil Analysis

The level of plant “greenness” was carried out using a soil and plant analyzer development (SPAD) meter (Minolta SPAD-502; Minolta, Osaka, Japan). This equipment allows the evaluation of the chlorophyll index of plant leaves without damaging the leaf. SPAD index was evaluated in all leaf stages from the cotyledons to the last developed plant stage.

At the seventh DAT, two plants shoots were harvested. At 21st DAT, the remaining plant was harvested and all roots recovered from the soil. Plant tissues were washed with 0.1% hydrochloric acid (HCl) and 0.01% of a nonionic detergent solution (Tween 80, Probus, Barcelona, Spain) to remove inorganic contaminants and dirt and subsequently rinsed twice with distilled water. Then, leaves, stems, and roots were separated and dried in a forced-air oven at 60°C for 72 h. Once dried, plant parts were weighted for the dry mass determination and calcinated at 480°C. Iron concentration was analyzed by AAS-FA, as described above, after acid digestion of the resulting ashes with HNO_3_ (Merck).

The remaining soil in each pot was also analyzed for Fe speciation, namely, the soluble and available Fe concentrations. For this purpose, two subsequent extraction steps were undertaken. First, the aqueous soluble Fe fraction was extracted: the total soil content of the pot was submerged and mixed with 600 mL distilled water and shaken for 10 min. Then, 40 mL of the mixture was centrifuged, and the supernatant was filtered (20−25 µm pore size filter) and acidified with HNO_3_. Second, the available Fe was extracted: 25 ml of an extractant solution [diethylenetriaminepentaacetic acid (DTPA) and ammonium bicarbonate] (Soltanpour and Schwab, 1977) was added to the soil that remained after the previous extraction, and the mixture was shaken in an orbital shaker (180 rpm, 10 min). After centrifugation, the supernatant was filtered through a filter paper (20–25 µm pore size) and recovered. This procedure was repeated three times, and the extracts were joined. Enough concentrated HNO_3_ was added to neutralize the present ammonium bicarbonate and, then, to acidify the solution; the final volume was set to 100 mL. For all samples, the concentration of Fe was analyzed as described previously.

### Data Statistical Analysis

All solution and soil interactions of ISS experiments were conducted at least twice, independently, with three replicates each, for a total of at least six samples. For soybean response to ISS application, each set was composed of five replicates. The SPAD indexes were read in triplicate for each leaf stage, in all five replicates, for a total of up to 15 (or 45, for measurements before the seventh day after treatment) measurements per level and treatment. Unless stated otherwise, data are presented as mean ± standard deviation.

The statistical analysis was performed using SPSS software (Ver. 25, SPSS Inc., Chicago, USA). When comparisons were required, an analysis of normality was first conducted using the Shapiro–Wilk test (a 99% level of confidence was used), followed by a Levene test of homogeneity of variance (95% confidence level). When normality and homogeneity of variance assumptions were verified, an ANOVA test was conducted to assess the significance of differences. A post-hoc test was also used, either a Dunnett’s *t* test (>negative control) or a Tukey’s honestly significant difference (HSD) test (95% confidence level), to compare differences to the negative control or to create subgroups of samples, respectively. Otherwise, when the two assumptions failed, the nonparametric Kruskal–Wallis test was conducted with a post-hoc pairwise comparison for subgrouping of sample sets.

## Results

### Calcareous Soil Interactions

The stability of ISS Fe chelates in the presence of Ca(II) (as a solution of 50 mmol L^−1^ CaCl_2_) was evaluated at different pH values. The media obtained from *A. vinelandii* exhibited greater stability at 3 days than the one from *B. subtilis*. In the case of *A. vinelandii*, ∼60% of the initial Fe remained solubilized, while <10% was found for *B. subtilis*. For each bacterial ISS, there were no substantial differences in the Fe-chelate stability efficiency within the pH range tested (**Table 1**).

**Table 1:** Influence of the pH on the stability of the iron-siderophore solution (ISS) Fe chelates.

Bacterial origin of ISS	Fe solubilized (%)^a^ vs. pH				
	7.0	7.5	8.0	8.5	9.0
*A. vinelandii*	64.7 ± 6.2	59.2 ± 5.2	60.0 ± 3.9	56.2 ± 4.8	61.8 ± 5.2
*B. subtilis*	4.6 ± 0.5	4.7 ± 1.5	5.0 ± 1.3	5.5 ± 2.1	6.6 ± 5.6

a The amount of Fe in solution was measured after 3 days of equilibrium and expressed in percent relative to the initial Fe added. The data correspond to the mean values and standard deviations of two independent experiences performed in triplicate (n = 6).

To have more clues regarding the interaction and properties of both media with soil, ISS of *A. vinelandii* and *B. subtilis* were tested in solution with soil and with the main components of the soil (**Table 2**). Again, both media have shown a different outcome. Whereas *A. vinelandii* ISS has shown higher quantities of Fe(III) still in solution, *B. subtilis* ISS showed higher losses at the end of the 3 days experiment. This behavior was visible with the soil components as well as with the soils. In addition, higher losses of soluble Fe were found in wet conditions for *B. subtilis*’ ISS, compared to *A. vinelandii’*s ISS: about 37 and 17% of initial Fe was recovered in Picassent soil for *A. vinelandii* and *B. subtilis* ISS, respectively, while about 41 and 9% were recovered for the standard soil.

**Table 2:** Influence of soil and soil components on the stability of ISS Fe chelates.

*Solid phase (in solution)*	Bacterial origin of ISS	
	*A. vinelandii* Fe solubilized (%)^a^	*B. subtilis*
*Solid phase (wet condition)*		
Ca-montmorillonite-	50.7 ± 4.1	8.7 ± 5.3
Peat	77.1 ± 5.3	50.4 ± 10.7
Goethite	99.2 ± 7.0	38.8 ± 9.6
Ferrihydrite	78.8 ± 9.8	80.0 ± 7.4
Calcium carbonate	92.5 ± 6.5	13.4 ± 5.4
Picassent soil	53.3 ± 12.5	10.8 ± 3.5
Standard soil	31.2 ± 3.6	9.4 ± 4.7
Picassent soil	36.9 ± 7.9	16.6 ± 6.3
Standard soil	41.5 ± 17.6	8.7 ± 3.0

a The amount of Fe in solution was measured after 3 days of equilibrium and expressed in percent relative to the initial Fe added. The data correspond to the mean values and standard deviations of two independent experiences performed in triplicate (n = 6).

### Application of Iron Siderophore Solution in Calcareous Soil and Soybean Response

#### Dry Plant Weight Evaluation

At the moment of sampling, plants were sectioned into their different main organs, namely, leaves, stems, and roots (these last only at the final sampling at 21 DAT). Then, after drying, plant parts were weighted (**Table 3**).

**Table 3:** Dry weight (g plant^−^^1^) of the different plant organs versus different treatments*^a^*.

Treatment	Plant organ	Days after treatment		Difference^b^
		7	21	
No Fe (C-)	Leaves	0.38 ± 0.05	0.46 ± 0.16	0.27 ± 0.15
	Stems	0.30 ± 0.05	0.36 ± 0.11	0.21 ± 0.10
	Roots	–	0.58 ± 0.25	–
	Shoots	0.68 ± 0.10	0.82 ± 0.27	0.48 ± 0.24
	Total	–	1.40 ± 0.51	–
*o,o*-EDDHA (C+)	Leaves	0.36 ± 0.10	0.62 ± 0.10	0.44 ± 0.15
	Stems	0.24 ± 0.08	0.41 ± 0.08	0.29 ± 0.11
	Roots	–	0.56 ± 0.10	–
	Shoots	0.60 ± 0.17	1.03 ± 0.18	0.73 ± 0.25
	Total	–	1.59 ± 0.26	–
*A. vinelandii*	Leaves	0.38 ± 0.16	0.60 ± 0.09	0.40 ± 0.14
	Stems	0.27 ± 0.09	0.42 ± 0.06	0.27 ± 0.09
	Roots		0.63 ± 0.08	
	Shoots	0.65 ± 0.25	1.02 ± 0.14	0.67 ± 0.22
	Total	–	1.66 ± 0.20	–
*A. vinelandii* (2)^c^	Leaves	–	0.62 ± 0.10	0.44 ± 0.14
	Stems	–	0.40 ± 0.05	0.28 ± 0.06
	Roots	–	0.67 ± 0.04	–
	Shoots	–	1.02 ± 0.14	0.72 ± 0.20
	Total	–	1.69 ± 0.15	–
*B. subtilis*	Leaves	0.47 ± 0.17	0.44 ± 0.05	0.19 ± 0.14
	Stems	0.28 ± 0.06	0.33 ± 0.03	0.20 ± 0.02
	Roots	–	0.55 ± 0.13	–
	Shoots	0.75 ± 0.21	0.77 ± 0.08	0.39 ± 0.15
	Total	–	1.31 ± 0.19	–
*B. subtilis* (2)^c^	Leaves	–	0.50 ± 0.11	0.28 ± 0.09
	Stems	–	0.33 ± 0.06	0.18 ± 0.06
	Roots	–	0.46 ± 0.10	–
	Shoots	–	0.83 ± 0.16	0.46 ± 0.12
	Total	–	1.29 ± 0.25	–

a The data correspond to the mean values and standard deviations of at least five plants measured in triplicate (n = 15).

b Average difference of dry mass in plants from 7th day until 21st day.

c Treatments with (2) represent plants with a second application performed 15 days after starting the treatment.

Data from the sampling conducted on the seventh day after treatment (7 DAT) shows no significant difference between the various treatments and the negative control. The stem average weights were particularly even. The leaves’ average values were even between controls and *A. vinelandii* ISS-treated plants, while *B. subtilis* ISS-treated plants had slightly higher dry weights. However, the scenario changed radically by the 21st day after treatment (21 DAT). Although again, no statistically significant differences (*p* > 0.05) in weight were found, two sets of data can be identified: *o,o*-EDDHA and *A. vinelandii* ISS-treated plants vs. negative control and *B. subtilis* ISS-treated plants. At 21 DAT, in the first group, the values measured for leaves, stems, and shoots were around 0.6, 0.4, and 1.0 g, respectively. However, roots on average had higher dry weights on *A. vinelandii* ISS-treated plants than on *o,o*-EDDHA ones. On the other hand, the second group averaged at about 0.4, 0.3, and 0.7 g for leaves, stems, and shoots, respectively.

**Figure 2:**
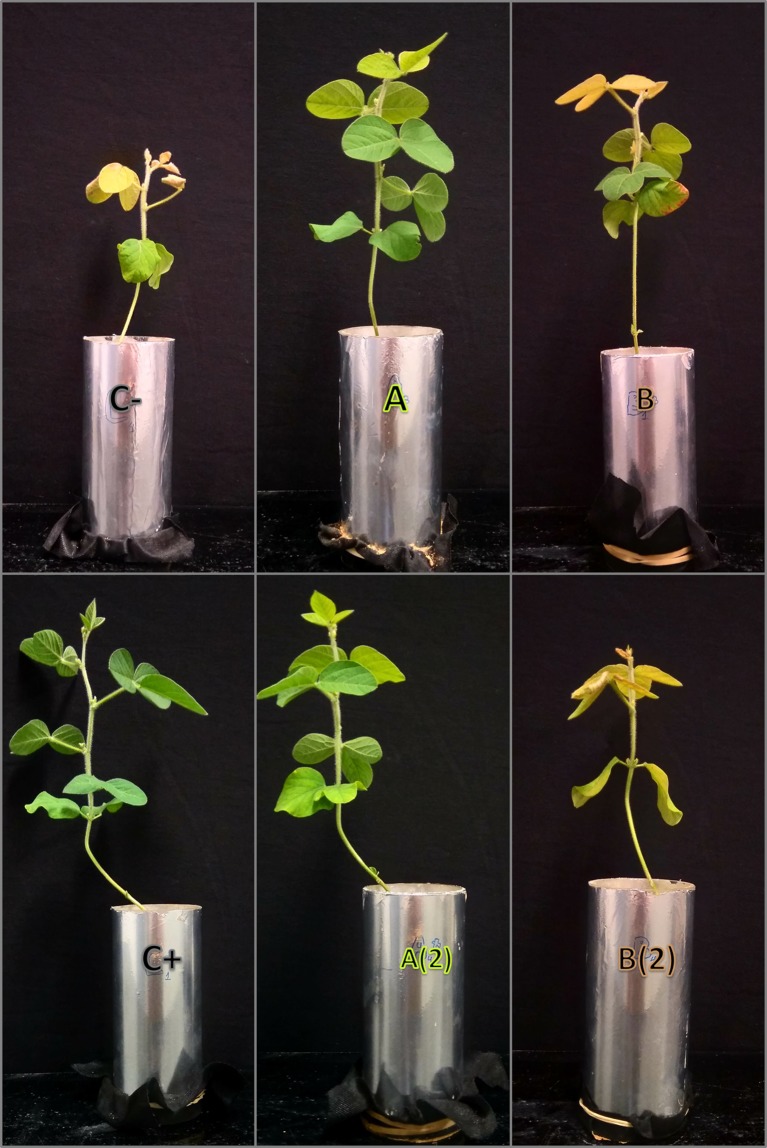
Photographs of representative plants at the 21st day after treatment (21 DAT). Negative control **(C−)**; positive control (C+; *o,o*-EDDHA); *A. vinelandii* ISS **(A)** and *Bacillus subtilis* **(B)** ISS. **A(2)** and **B(2)**: second application of *A. vinelandii* ISS **(A)** and *Bacillus subtilis* **(B)** ISS, respectively, at 15th day after treatment (15 DAT).

The mass plant development can be seen when calculating the difference between 7 and 21 DAT plant mass. On average, leaves, stems, and shoots of the plants treated with *o,o*-EDDHA, and *A. vinelandii* ISS have grown more 50, 33, and 43%, respectively, than the negative control and *B. subtilis* ISS-treated plants. Moreover, 21 days after treatment application, plants supplied with Fe-*A. vinelandii* ISS or Fe-o,o-EDDHA fertilizers were greener than the negative control (**Figure 2**). On the other hand, plants of the negative control evidenced chlorosis and had a significantly impaired plant development (**Figure 2**).

#### Soil and Plant Analyzer Development

**Figure 3:**
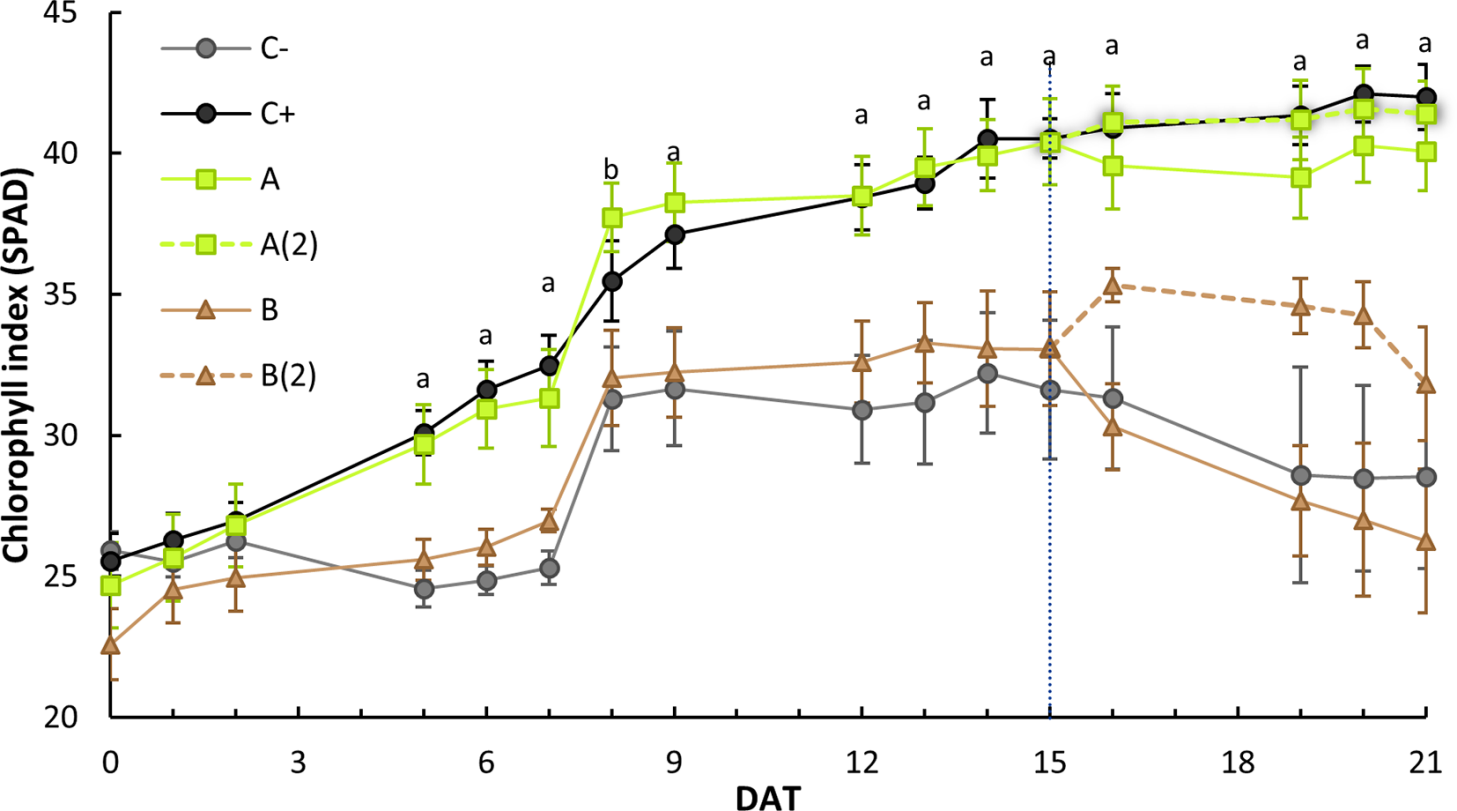
Stage 2 leaves average and standard error of the soil and plant analyzer development (SPAD) index evolution on the course of the 21 days after treatment(s) (DAT). At 15 DAT, a second treatment was applied to half of A and B plants [designated as A(2) and B(2), respectively], splitting both sets (*n* = 10) into two sets (*n* = 5). Each plant SPAD was read in triplicate. Different letters denote a significant difference of SPAD levels within each treatment (95% confidence interval), as shown by Dunnett’s *t* test (> negative control): a = both *o,o*-EDDHA (C+) and *A. vinelandii* ISS-treated plants [A and A(2)] were statistically different (*p* < 0.05) than the negative control (C−); b = Only *A. vinelandii* ISS-treated plants were statistically different (*p* < 0.05).

The evolution of plant “greenness” was evaluated using the chlorophyll meter SPAD in all leaf stages after the cotyledons to the last developed plant stage. The results for plant stage two can be seen in **Figure 3**. This stage was chosen because all plants had fully developed this stage at the start of the trial (0 DAT) and had relatively similar initial values between treatments; therefore, a better comparison of the treatment effect is achievable.

As can be seen in **Figure 3**, plants treated with *o,o*-EDDHA and *A. vinelandii* ISS developed higher levels of chlorophyll than those of the negative control and plants treated with *B. subtilis* ISS. An ANOVA analysis revealed that this difference was significant (*p* < 0.05) from the eighth day after treatment (8 DAT) onward until the end of the experiment. A Dunnett’s *t* post-hoc test also showed that, for all days after 8 DAT, *o,o*-EDDHA and *A. vinelandii* ISS-treated plant sets were statistically different (*p* < 0.05) than the negative control (**Figure 3**).

**Figure 4:**
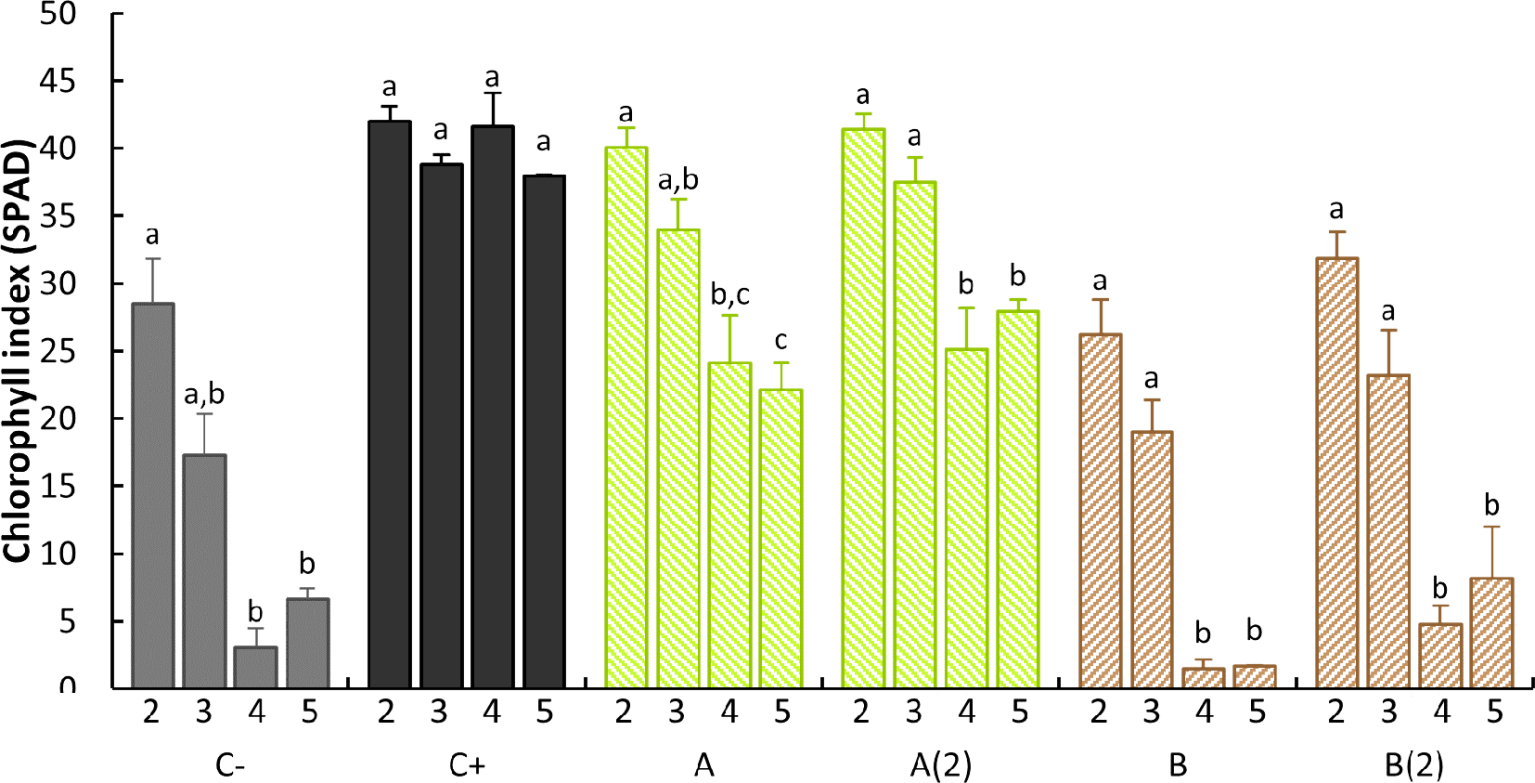
Intratreatment leaf stage comparison of the SPAD average read and standard error at 21 DAT. Different letters denote a significant difference of SPAD levels within each treatment (95% confidence interval) as shown by Tukey’s HSD test. For intraleaf stage treatment comparison, please check **Figure S1** in the supplementary material. C−: no iron treatment (negative control); C+: *o,o*-EDDHA (positive control); **(A)** *A. vinelandii* ISS; **(B)** *B. subtilis* ISS. A(2) and B(2) represent plants with a second application performed 15th day after the first treatment (*n* = 5).

Further examination was conducted on data from 21 DAT, which allowed to examine the influence of each treatment on each stage level. The analysis of **Figure 4** allows us to draw three different patterns: *o,o*-EDDHA’s (positive control) leaf stages showed no statistically significant differences within all leaf stages; the negative control and both *B. subtilis* ISS-treated plants had a sharp decrease from the third to the fourth leaf stages, whereas the fifth stage remained equally low as forth; both *A. vinelandii* ISS-treated sets had a moderate decrease in SPAD from third to fourth/fifth leaf stages (**Figure 4**).

#### Fe Content in Soybean and Soil Fe Speciation

**Figure 5:**
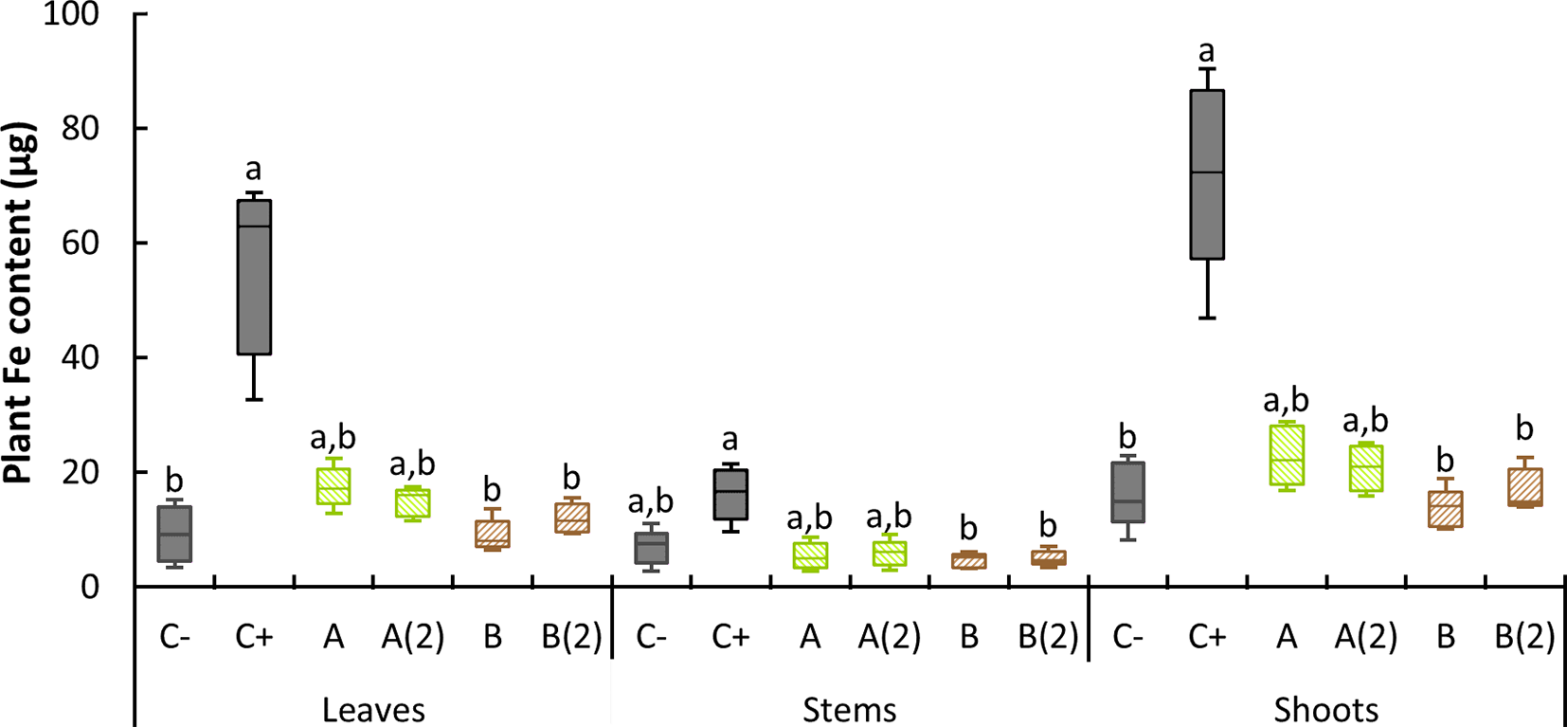
Boxplot representation of Fe content per plant found at 21 DAT. C−: no iron treatment (negative control); C+: *o,o*-EDDHA (positive control); **(A)** *A. vinelandii* ISS; **(B)** *B. subtilis* ISS. Different letters denote a significant difference of Fe content levels for each organ within a 95% confidence interval, as shown by the pairwise comparisons of the nonparametric Kruskal–Wallis test (*n* = 5).

Dry plant matter was subjected to calcination, and the resulting ashes were digested in HNO_3_. Metal content was subsequently measured. Data from Fe content in leaves, stems, and shoots, per plant, are presented in **Figure 5**.

Plants treated with *o,o*-EDDHA evidenced the highest Fe content among all treatments, within both leaves and stem. Conversely, on leaves, both negative control and *B. subtilis* ISS-treated plants showed the lowest amount of Fe. In stems, all but *o,o*-EDDHA, were equally low. Using the pairwise comparisons of the nonparametric Kruskal–Wallis test (since no homogeneity of variance was found in the samples), significant differences were found between *o,o*-EDDHA and negative control in leaves and shoots. In addition, using the same test, differences in all tested tissues between both *B. subtilis* samples and *o,o*-EDDHA were found. From this pairwise comparison, two subsets always resulted: one subset containing *o,o*-EDDHA and another one containing negative control and *B. subtilis*. *A. vinelandii* samples were placed in both subsets, as it can be seen in **Figure 5**.

The analysis of the soil Fe speciation may shed some clues on the outcomes of treatments applied. Two fractions were analyzed: the water-soluble phase (WSP) and the DTPA extracted (available Fe) phase (DEP) (**Table 4**).

**Table 4:** Average of Fe concentration in soil (mg kg^−^^1^) in two different phases for different treatments.

Treatment	Fe concentration (mg kg^−1^)^a^	
	WSP	DEP
No Fe (C−)	0.03 ± 0.04 b	7.08 ± 0.70 c
*o,o*-EDDHA (C+)	0.68 ± 0.03 a	6.80 ± 0.41 c
*A. vinelandii*	0.02 ± 0.04 b	7.29 ± 0.91 c
*A. vinelandii (2)*	0.01 ± 0.03 b	8.41 ± 0.82 b,c
*B. subtilis*	0.00 ± 0.03 b	10.58 ± 2.39 b
*B. subtilis (2)*	0.00 ± 0.04 b	15.70 ± 2.11 a

a Water-soluble phase (WSP) and DTPA extraction phase (DEP). Different letters denote a significant difference on Fe concentration within 95% confidence interval, as shown by Tukey’s HSD test. Treatments with (2) represent plants with a second application performed 15th day after the first treatment. Average ± standard deviation (n = 5).

For WSP, significant amounts of Fe were found only in the case of soils treated with *o,o*-EDDHA, with an average of 0.68 mg kg^−1^. All other soils had very limited or null concentrations found (**Table 4**). On the other hand, for DEP, in both controls and *A. vinelandii* ISS-treated soils, low amounts of Fe were extracted (between 6.80 and 8.41 mg kg^−1^). Statistical analysis has shown that, in WSP conditions, all samples but *o,o*-EDDHA were grouped, while in DEP, *o,o*-EDDHA, *A. vinelandii*, and negative control are grouped with the lower quantity of Fe found, whereas both *B. subtilis* conditions were each separated with higher amounts of available Fe.

## Discussion

### Calcareous Soil Interactions

As a first step, the suitability of the ISS was evaluated for IDIC amendment, and their properties were studied by performing solution and soil interaction studies. These assays provide an idea of how effective (or ineffective) a Fe-chelating solution agent might be (Álvarez-Fernández et al., 1997). They may also provide information about possible interactions, which may occur in the calcareous soils (López-Rayo et al., 2014). These data are potentially useful for predicting the outcome in field application as well as will help in understanding the results obtained latterly.

#### ISS Stability for Soil Application

It is possible to see that some or most Fe have precipitated in *A. vinelandii* and *B. subtilis* ISS, respectively (**Table 1**). In a previous work (Ferreira et al., 2019b), it was verified that the siderophores produced by both bacteria evidenced high affinity for Fe, especially at neutral to alkaline pH. Thus, based on our previous results, a higher Fe complexation efficiency (namely, with *B. subtilis* ISS) would be expected in the presence of Ca(II) solution. The fact that part or most of Fe precipitated after 3 days (**Table 1**) may be due to one, or a combination, of the following reasons: 1) the simple inability of the ISS Fe chelates to compete with the considerable amount of Ca(II) ions present in solution; 2) degradation of the Fe chelates over time; 3) the presence of a mix of different Fe chelates with different stabilities; 4) coprecipitation phenomena that took out Fe from the solution.

The first hypothesis seems to be unlikely. The literature describes the values of the Fe-chelate constants for the main siderophores produced by *A. vinelandii* and *B. subtilis*. For example, in the case of protochelin, the tri-catecholate siderophore produced by *A. vinelandii*, the Fe formation constant is 10^43.9^, identical to that of ferrioxamine B (Cornish and Page, 1998), whereas, for bacillibactin, the tri-catecholate produced by *B. subtilis*, the Fe formation constant is equally high (10^47.6^) (Dertz et al., 2006). To our knowledge, no formation constants for any of these siderophores with Ca(II) were described; therefore, a theoretical analysis of the competition of Ca(II) for the binding sites is not possible. Nonetheless, the catecholate moieties are very selective to hard trivalent metal ions (Hider and Kong, 2010), such as Fe(III), relatively to divalent ions as is the case of Ca(II). The decrease in selectivity from trivalent to divalent ions is notoriously visible by the reduction in the formation constants values from Fe(III) to Fe(II) (Hider and Kong, 2010). Based on all these facts, the Ca(II) competition in solution should not be a major deterring factor.

On the other hand, the second possibility is somewhat feasible: siderophores, as natural products, are likely to be biodegradable. In addition, the abiotic degradation of siderophores has been reviewed (Harrington et al., 2015). The factors usually involved in abiotic degradation, such as radiation exposure or high temperatures, were prevented. However, and in particular, in the cases where no low molecular weight organic acids were present, reactions at the surface of soil components, especially in the Fe minerals, may result in the degradation of coordinating moieties with consequent loss of Fe (Harrington et al., 2015). Therefore, the degradation by biotic or abiotic processes is a possibility and may explain part of the decrease in the Fe(III) chelation efficiency. However, the considerable differences between the two ISS are unlikely related to this phenomenon. In a previous work (Ferreira et al., 2019b), the analysis of the culture filtrates of *A. vinelandii* and *B. subtilis* revealed the presence of catechol-type siderophores, which is compatible with the possible presence of several siderophores described in the literature for each bacterium, namely azotobactin and protochelin for *A. vinelandii* and bacillibactin for *B. subtilis* (Ferreira et al., 2019a).

The third reason is also plausible since other different Fe-chelating molecules may be present at various quantities. Different media compositions are expected due to the various culture media initially used for growing the bacteria as well as different exudates produced by the bacteria. Different mild Fe-chelating agents may be present, which are capable of complex Fe(III). One such example may be gluconic acid, resultant from the activity of glucose dehydrogenase, produced during the sporulation of *B. subtilis* (Fujita et al., 1977), which is known to complex Fe(III) (Sawyer, 1964). However, the resulting complexes are likely weak and may lose their capacity when disturbed by the addition of high concentrations of Ca(II).

The occurrence of coprecipitation as a cause of Fe loss is also possible. In fact, during the experimental procedure, it was possible to see that upon the addition of Ca(II) to the *B. subtilis* ISS, a white-reddish precipitate was formed, and a change in solution hue and saturation was also observed, indicating the precipitation of the iron previously present in solution. In *A. vinelandii* medium, such behavior was not found. Different concentrations of phosphate in ISS, as a result of different initial phosphate concentrations used in the two different growth media (higher in *B. subtilis* than in *A. vinelandii*), may explain the distinct results. The presence of different organic exudates may also help to explain the discrepancies for the two ISS.

Regarding the interaction of ISS with soils and its components (**Table 2**), the behavior of both ISS in the presence of CaCO_3_ mimics the profile recorded in the previous experiment (**Table 1**). While in the case of *A. vinelandii* medium, an average of 92% of the Fe initially added was recovered at the end of the experiment, in the case of *B. subtilis* medium, only 13% of Fe was recovered. The lower precipitation of Fe observed for both ISS, compared to the previous experiment, may be related to the lower concentration of Ca(II) in solution. While in the last experiment, CaCl_2_ was dissolved to 50 mmol L^−1^, the latter was conducted in the presence of less amount of soluble CaCO_3_. Based on the solubility product of CaCO_3_ (10^−8.48^) (Martell and Smith, 2004), the concentration of free Ca soluble under these conditions should be under 5 mmol L^−1^, which is substantially lower than the value previously used (50 mmol L^−1^). Therefore, it is plausible that the higher precipitation previously observed on *B. subtilis* ISS is correlated with the formation or presence of inorganic Ca precipitates, such as calcium phosphates, or other inorganic/organic compounds as seen previously for Mn chelates (López-Rayo et al., 2014). For both solutions, Ca-montmorillonite was the soil component that evidenced the lower amount of Fe(III) solubilized (**Table 2**): at the third day, 50.7 and 8.7% in the case of *A. vinelandii* and *B. subtilis* ISS, respectively. This observation is in agreement with the observations that siderophores produced by microorganisms are adsorbed in the clays (Siebner-Freibach et al., 2004). The substantial difference of adsorption between both may be due to the charge and hydrophobicity of the siderophore(s) present in each ISS (Crowley, 2006) and/or the difference of ionic composition of the media, which was shown to influence the adsorption of siderophores to Ca-montmorillonite (Siebner-Freibach et al., 2004). Regarding the Fe (oxy)hydroxides tested, the interaction of ferrihydrite with both media had similar results (∼80% of Fe remained soluble at the end of the third-day experiment). In contrast, goethite had different behaviors in the two ISS: for *A. vinelandii* ISS, an average of 99% Fe was found in solution, while only 39% was found in the case of *B. subtilis* ISS. For some samples, the Fe dissolved content at the end of the experiment was higher than in the beginning. These substantial differences may be the result of two actions taking place: adsorption of siderophores to the mineral’s surface and promotion of mineral dissolution by siderophores and other organic acids present in solution (Kraemer, 2004). The presence of organic acids can also be an important factor in the dissolution of goethite (Vinet and Zhedanov, 2011).

In a previous work, similar tests were carried out with two synthetic siderophores, one being azotochelin, a catecholate siderophore produced by *A. vinelandii* (Ferreira et al., 2019c). In it, Fe-azotochelin chelates had lower interaction profiles with soil components and with soil, and thus, Fe-azotochelin chelates remained dissolved. Regarding the stability of Fe(III) in the presence of Ca(II) solution, our results are in fair agreement with those obtained for Fe(III)-azotochelin solution. Azotochelin evidenced lower interactions with the different soil components than *A. vinelandii*’s ISS. The differences found may be attributed to a different siderophore composition and/or the presence of other organic and inorganic compounds in solution. The variances in the siderophore composition can justify some differences in the adsorption profiles; however, the presence or absence of other organic molecules explains better the discrepancies. For example, ferrihydrite is more soluble than goethite and more easily dissolved by siderophores when acting alone (Kraemer, 2004), whereas goethite dissolution may be improved by the presence of organic acids (Vinet and Zhedanov, 2011), which would justify the increase in *A. vinelandii* ISS case. On the contrary, the presence of different electrolytes in *A. vinelandii* ISS (and the absent in azotochelin study) may be a key element in stabilizing the adsorbed siderophores on the other solid phases tested.

Compared to other synthetic chelating agents, such as *N,N’*-bis(2-hydroxyphenyl)ethylendiamine-*N,N’*-diacetic acid (HBED), *N,N’*-bis(2-hydroxy-5-methylphenyl)ethylendiamine-*N,N’*-diacetic acid (HJB), or *o,o*-EDDHA (López-Rayo et al., 2009), both *A. vinelandii* ISS and particularly *B. subtilis* ISS underperform under similar conditions, since these ISS formulations showed little to no interactions with the soil components or mobilization in the pH range tested in this work. However, compared to other natural-based chelating agents, namely, lignosulfonates, amino acids, gluconates, and humates (Lucena et al., 2010), and despite the slightly worse stability that *A. vinelandii*’s ISS evidenced in Ca(II) alkaline solutions, these compounds underperform when in contact with soils or soil components compared to *A. vinelandii*’s ISS.

#### Chelate Stability in Soil

With the purpose to mimic the soil irrigation under agronomic conditions, a smaller volume of a chelate solution may be applied to simulate soil experiments. Many authors considered this setup as an ideal experimental setting (Limousin et al., 2007; Hernández-Apaolaza and Lucena, 2011). The lower solution/soil ratio is preferred because it allows better observation of the chelates retention, which is difficult to study when high solution/soil ratios are used (Hernández-Apaolaza and Lucena, 2011). The results obtained for both bacterial ISS can be seen in **Table 2** [see Solid phase (wet condition)].

The results obtained with this setup mimic those found for the solution experiments described earlier. Recently, similar tests were conducted with Fe-azotochelin solutions, prepared from chemically synthetized azotochelin (Ferreira et al., 2019c). Some differences in the total amount of soluble Fe(III) at the end of the 3 days were visible in the different tests conducted in solution and wet conditions, as well as between the two soils tested (standard soil vs. Picassent soil). Contrary to these findings, here in this study, not only no substantial difference in the amount of soluble Fe(III)-chelates between both soils under test was found for *A. vinelandii* ISS but also no significant decrease was observed when moving to a more intimate soil interaction (**Table 2**). For *B. subtilis*, the results obtained for this experiment are also in good agreement with those obtained with the previous solution experiments. Since comparable amount of soluble Fe(III) was registered in both experiments [in **Table 2**, Solid phase (in solution) and Solid phase (wet condition), respectively], the results from both ISS seem to indicate that no significant changes in the adsorption profile occurs when the solution/soil ratio is changed relative to the experiments with solid-phase in solution.

##### Solid Phase (In Solution)

The higher retentions on the standard soil may be a result of the finer texture of the soil and high clay composition. Despite the low Fe recovery results, the average Fe concentration found in the soil solution in all experiments was found to be in the range between 10^−4^ and 10^−5^ mol L^−1^. These concentrations can be considered suitable for soil application since they are within the required Fe concentration range described for plants (10^−4^ to 10^−9^ mol L^−1^) (Kim and Guerinot, 2007).

In conclusion, from these solution and soil interactions tests with the ISS, both ISS behaved differently: *A. vinelandii* ISS was more stable and maintained more Fe in solution than *B. subtilis*, keeping ∼40% of the initial Fe in wet soil conditions. The latter showed a higher degree of adsorption and soluble Fe loss, with more than 85% of initial Fe lost under the same conditions. However, both were able to maintain Fe in a concentration range adequate for plant development (10^−4^ to 10^−9^ mol L^−1^) (Kim and Guerinot, 2007).

### Application of Iron Siderophore Solution in Calcareous Soil and Soybean Response

#### Soil and Plant Analyzer Development

The results found for the SPAD of stage 2 leaves along the 21 days after the treatment (**Figure 3**) are comparable to the SPAD data obtained by Martins et al. (2018) and Ferreira et al. (2019c) when synthetic siderophores [Fe(III)-azotochelin] or analogs thereof [Fe(III)-*N*,*N*′-dihydroxy*N*,*N*′diisopropylhexanediamide] chelates were tested as Fe sources to cucumber plants in hydroponic conditions and soybean (*G. max*) in calcareous soils, respectively. In addition, when examining the data from the SPAD of the 21 DAT (**Figure 4**), it is possible to observe that, unless for *o,o*-EDDHA, all other treatments had shown a decrease in the effectiveness over time, in particular, *B. subtilis* ISS. With the use of *o,o*-HBED and *o,o*-EDDHMA Fe chelates, similar SPAD profiles to those of *A. vinelandii* ISS were obtained for the last fully developed trifoliate 28 DAT (Bin et al., 2016). In this study, Bin et al. (2016) reported that both Fe(III)-HBED and Fe(III)-EDDHMA chelates performed good, with just slightly lower performance than *o,o*-EDDHA. On the contrary, the effectiveness of *B. subtilis* ISS was comparable to the one registered for the negative control.

Moreover, the intrastage treatment statistical analysis, presented in **Figure S1**, upholds the previous assumption. Consistently, *B. subtilis* ISS-treated plants were grouped with the negative control in all stages, whereas *A. vinelandii* ISS-treated plants were paired with *o,o*-EDDHA set in the second and third stages and arranged alone in the fourth and fifth stages (**Figure S1**). A typical result for both bacterial ISS corresponds to the apparent loss of Fe provision efficiency over time (*B. subtilis* much more than *A. vinelandii*). In the data obtained by Yehuda et al. (2000) for rhizoferrin Fe chelates in a nutrient solution, it was possible to observe a relationship between chelate concentration and a decrease in SPAD readings in latter leaf stages. The results obtained in our study, in particular in *B. subtilis* ISS, may be due to biodegradation of the siderophore(s) and/or insufficient Fe provided in the solution. Conversely, the *o,o*-EDDHA exerts its effect throughout the entirety of the trial due to its persistence, whereas possible degradation decreases the effect of bacterial ISS over time, in particular in *A. vinelandii* ISS, as it appears to be effective in the early stages of the experiment. Comparing *A. vinelandii* ISS results with those of *B. subtilis’*, the first evidenced to be more stable than the last. Besides the possible biodegradation of the siderophore(s), soil adsorption phenomena may also contribute to decreasing the availability of Fe siderophores in both cases; in fact, previous experiments have demonstrated high levels of Fe losses to the soils in the case of *B. subtilis* ISS, while less Fe adsorption was recorded for *A. vinelandii* ISS (**Table 2**). On a final note, the second application of ISS has resulted in little effect. Schenkeveld et al. (2010) have demonstrated that when Fe chelates (*o,o*-EDDHA in their case) were applied to chlorotic plants, some time was needed for SPAD values to increase significantly. Therefore, the lack of effect of the second application may be due to the short time that plants were allowed to recover from chlorosis (Schenkeveld et al., 2010).

The SPAD results (**Figure 3**) are in agreement with those obtained for plant dry mass (**Table 3**). Therefore, the comparable development of plants treated with *o,o*-EDDHA and those treated with *A. vinelandii* ISS lead us to conclude that *A. vinelandii* ISS had a positive effect on the plant development and chlorosis amendment. On the other hand, the comparable behavior of *B. subtilis* ISS-treated plants with the one recorded for the negative control, evidenced that *B. subtilis* ISS had no significant effect on the plant growth of chlorotic soybean plants. This fact might be due to the effect of Fe chelates in *A. vinelandii* ISS being able to deliver iron to soybean plants during the first days after application. Therefore, an accumulation of enough Fe in the early developed and developing leaves to allow chlorophyll production in these leaves occurred. However, sometime after the application, Fe chelates in *A. vinelandii* ISS became less efficient (possibly due to degradation), and the Fe uptake decreased. The newly developed leaves had a lower Fe supply and developed less green (see **Figure S2** and the following section for clarification).

#### Fe Content in Soybean and Soil Fe Speciation

Considering the dry mass (**Table 3**) and chlorophyll content (**Figure 3**) results, the plant Fe values found in *A. vinelandii* ISS (**Figure 5**) seems particularly low when compared with those obtained for *o,o*-EDDHA. At a median of ∼20 µg of Fe per plant, they were lower than previously reported for the siderophore azotochelin synthetically produced in the lab (Ferreira et al., 2019c). Considering that the effectiveness of siderophores in providing Fe to strategy 1 and 2 plants has been demonstrated before (although for shorter periods of time) (Yehuda et al., 1996; Chen et al., 2000), it is unlikely that the reason for the lower amount of Fe in plants was due to incompatibility of siderophores with plants. Given the evolution of the SPAD values registered in the different plant growth stages (**Figure 4**), one possible explanation can be that latter leaf stages had less Fe content than the first ones (see **Figure S2** for clarification). Since all the leaf dried mass was ground and homogenized, the low content of Fe present in the upper part of the plant (leaves and shoots) decreased the overall leaf and shoot Fe content. Nonetheless, the initial quantities of Fe taken by the plant were sufficient for the initial recovery of soybean plants and chlorophyll production. A ratio between Fe content determined in leaves and the stem was calculated (**Table 5**) to obtain information about the translocation of Fe from the stem to leaves. Higher values of the translocation ratios can be related to faster Fe uptake by leaves: as Fe moves from the transport organ (stem) into leaves, less is found in the first and more in the latter, increasing the ratio value (Bauer and Hell, 2006). On average, *o,o*-EDDHA presented the highest ratio, followed by the values found for *A. vinelandii* and *B. subtilis* treatments. Control negative showed the lowest value. The value found for *o,o*-EDDHA was higher than those obtained by Ferreira et al. (2019c) but of the same order of magnitude of those calculated from the data collected by Gonzalo et al. (2013). Using an ANOVA, followed by a Tukey’s HSD test, two sets were found: plants with high translocation ratios [*o,o*-EDDHA, A, A(2) and B(2)] and those with lower translocation ratios [C−, A(2), B and B(2)]. However, differences in translocation ratios within both *A. vinelandii* and *B. subtilis*-treated plants [A vs. A(2) and B vs. B(2)] were due to differences in leaf Fe content and not because of Fe content in the stem, as it remained similarly low (**Figure 5**). We can conclude that, in both cases, Fe translocation from stem to leaves occurs, and therefore, any Fe absorbed by roots shall, in theory, reach leaves easily. The *B. subtilis*-treated plants had a lower ratio in the result of the overall lower Fe uptake and consequent translocation of Fe to leaves.

**Table 5:** Average leaves Fe content to stem Fe content ratio in different treatments.

Treatment	Fe content_leaves_ / Fe content_stem_^a^
Without Fe (C−)	1.52 ± 0.72 b
*o,o*-EDDHA (C+)	3.72 ± 1.72 a
*A. vinelandii*	3.66 ± 1.43 a
*A. vinelandii* (2)	2.87 ± 1.15 a,b
*B. subtilis*	1.99 ± 0.55 b
*B. subtilis* (2)	2.48 ± 0.55 a,b

a Average ratio of Fe content found in leaves to Fe content found in stem for each treatment at DAT 21. Different letters denote a significant difference of ratio within 95% confidence interval, as shown by Tukey’s HSD test. Treatments with (2) represent plants with a second application at 15th day after first treatment.

The results found for the soluble soil Fe extraction are similar to those found recently by Martín-Fernández et al. (2017), where only *o,o*-EDDHA presented a high quantity of soluble Fe in the soil, whereas it was less for other biodegradable organic chelating agents tested. On the other hand, in DEP values, considering the value of the negative Fe control (C−), most Fe present likely resulted from the dissolution of Fe oxy-hydroxides, which were dissolved by DTPA solution. DTPA extractions are generally considered a good representation of metal availability to plants although with some limitations (Contin et al., 2007). However, given the similar concentrations found in DEP of A and A(2) to those of the negative control, there is no apparent correlation of DEP concentrations with the Fe intake or chlorophyll production. The slightly larger quantities found for *A. vinelandii* ISS-treated soils may be due to some adsorption phenomena of the added Fe chelates, as it was witnessed previously in the soil interaction assays. A significant different profile was observed with both *B. subtilis* ISS-treated soils, where higher quantities of Fe were extracted. Again, although higher amounts of Fe in DEP were found in soils treated with *B. subtilis* ISS, these did not translate in higher chlorophyll production or Fe intake. These differences are consistent with the proposition that *B. subtilis*’ siderophores (or Fe chelates thereof) adsorb to the soil phases and remain bio-unavailable. Furthermore, DEP concentrations found for all other cases are very likely linked to the presence of Fe oxy-hydroxides, which are not available for plants. Consequently, Fe found in this manner is not available and is not provided to plants, which lead for example on the development of chlorosis, as it was observed for *B. subtilis* ISS-treated soybean plants (**Figure 2**), with less Fe in tissues (**Figure 5**).

The ISS application to chlorotic soybean plants grown in calcareous soil has resulted in two different outcomes for each of the ISS tested: *A. vinelandii* ISS-treated plants had a dry mass and chlorophyll development similar to that of the positive control (*o,o*-EDDHA), while *B. subtilis* ISS-treated plants had a development similar to that of negative control. Iron intake was lower in both cases compared to positive control; however, in *A. vinelandii* case, it was higher than the negative control.

Although the effect of culture media components (like carbohydrates) or other compounds (for example, secondary metabolites produced during the bacterial growth) can affect the growth of plant roots, they should not play an important role in the context of the present work (correction of iron deficiency). Compatible with this possibility, similar dry weight values of the roots of soybean plants, quantified after 21 DAT, in the positive control (o,o-EDDHA) and treated with *A. vinelandii* or *B. subtilis* ISS were observed (**Table 3**), which indicate that the growth of roots were not affected (positively or negatively) by the presence of the remaining media components or possible metabolites. Regarding other elements present in the medium, as it was mentioned above, the major macronutrients present in each ISS solution were analysed, and at the moment of the application of ISS, the addition of appropriate solutions were applied in order to balance the quantities of macronutrients added (the same quantity of major nutrients was present in all pot sets) and thus eliminating the effects of additional nutrients that may occur.

## Conclusions

In this work, the ability of two new Fe freeze-dried fertilizer products, prepared from the filtrate cultures of *A. vinelandii* and *B. subtilis*, for amending IDIC of soybean plants grown in calcareous soils was evaluated.

Plants treated with *A. vinelandii* Fe fertilizer developed a dry mass comparable to that of *o,o*-EDDHA and a significant increase in the SPAD levels when compared to the negative control plants. Conversely, plants treated with *B. subtilis* iron fertilizer had a response (dry mass and SPAD) comparable to the one registered for the negative control. *A. vinelandii*-treated plants had higher Fe content than *B. subtilis*-treated plants. Therefore, higher efficiency for correcting IDIC was observed when *A. vinelandii* Fe fertilizer was used comparatively to *B. subtilis *one. Together, the results pointed out that the freeze-dried product, prepared from *A. vinelandii*, represents a very promising, sustainable, and environment-friendly Fe-fertilizer alternative for application in the IDIC amendment in calcareous soils.

## Data Availability Statement

All datasets generated for this study are included in the article/**Supplementary Files**.

## Author Contributions

HS, JL, and ES designed research. CF performed research under the supervision of HS, ES, and SL-R. HS, JL, CF, ES, and SL-R analyzed data. CF, HS, JL, and ES wrote the paper. All authors read and approved the final manuscript.

## Funding

This work was financially supported by Project PTDC-AGR-TEC-0458-2014—POCI-01-0145-FEDER-016681—funded by FEDER funds through COMPETE2020—Programa Operacional Competitividade e Internacionalização (POCI) and by national funds (PIDDAC) through FCT/MCTES and Project RTI2018-096268-B-I00 by the State Research Agency, Ministry of Science, Innovation and Universities of Spain.

## Conflict of Interest

The authors declare that the research was conducted in the absence of any commercial or financial relationships that could be construed as a potential conflict of interest.

## Abbreviations

AAS-FA, atomic absorption spectroscopy with flame atomization; APCAs, aminopolycarboxylic acids; BM, burk’s medium; DAT, days after treatment; DEP, DTPA extracted phase; DSM, deutsche sammlung von mikroorganismen und zellkulturen; DTPA, diethylenetriaminepentaacetic acid; HBED, *N*,*N*′-bis(2-hydroxyphenyl)ethylendiamine-*N*,*N*′-diacetic acid; HJB, *N*,*N*′-bis(2-hydroxy-5-methylphenyl)ethylendiamine-*N*,*N*′-diacetic acid; IDIC, iron-deficiency-induced chlorosis; ISS, iron-siderophore solution; MM, minimal medium; o,o EDDHA, ethylenediaminedi(o-hydroxyphenylacetic) acid; SPAD, Soil and plant analyzer development; WSP, Water-soluble phase.
