# Supplementary material for: Evaluation of the Efficacy of Two New Biotechnological-Based Freeze-Dried Fertilizers for Sustainable Fe Deficiency Correction of Soybean Plants Grown in Calcareous Soils
Source: Front Plant Sci. 2019 Nov 8;10:1335. doi: 10.3389/fpls.2019.01335 (PMC6857624; doi:10.3389/fpls.2019.01335)
Supplement: Supplementary file 1 [file DataSheet_1.docx]

FRONTIERS IN PLANT SCIENCE

Evaluation of the efficacy of two new biotechnological-based freeze-dried fertilizers for sustainable Fe deficiency correction of soybean plants grown in calcareous soils

*Supplementary Materials*

Carlos M.H. Ferreira^1,2,3^, Sandra López-Rayo^4^, Juan J. Lucena^4*^, Eduardo V. Soares^2,3^, Helena M.V.M. Soares^1*^

^1-^REQUIMTE/LAQV, Departamento de Engenharia Química, Faculdade de Engenharia, Universidade do Porto, rua Dr. Roberto Frias, 4200-465, Porto, Portugal

^2-^Bioengineering Laboratory-CIETI, Chemical Engineering Department, ISEP-School of Engineering of Polytechnic Institute of Porto, rua Dr António Bernardino de Almeida, 431, 4249-015 Porto, Portugal

^3-^CEB-Centre of Biological Engineering, University of Minho, Campus de Gualtar, 4710-057 Braga, Portugal

^4-^Departamento de Química Agrícola y Bromatología, Facultad de Ciencias, Universidad Autónoma de Madrid, 28049-Madrid, Spain

***Corresponding authors:**

[hsoares@fe.up.pt](mailto:hsoares@fe.up.pt) (Helena M.V.M. Soares);

[juanjose.lucena@uam.es](mailto:juanjose.lucena@uam.es) (Juan J. Lucena)

1. Culture media composition

*Bacillus subtilis* was kept in minimal medium (MM) agar while *Azotobacter vinelandii* was kept in Burk´s medium (BM) agar (Newton et al., 1953), at 4º C. MM agar contained per liter: 10 g glucose, 1.47 g glutamic acid, 3.0 g potassium hydrogenophosphate (K_2_HPO_4_), 1.0 g potassium dihydrogenophosphate (KH_2_PO_4_), 0.5 g ammonium chloride (NH_4_Cl), 0.1 g ammonium nitrate (NH_4_NO_3_), 0.1 g sodium sulphate (Na_2_SO_4_), 10 mg magnesium sulphate heptahydrate (MgSO_4_.7H_2_O), 1 mg manganese(II) sulphate tetrahydrate (MnSO_4_.4H_2_O), 0.5 mg calcium chloride (CaCl_2_) and 20 g agar. BM agar was prepared as previously described (HiMedia Laboratories, 2015) replacing sucrose by glucose; the medium contained per liter: 10 g glucose, 0.8 g K_2_HPO_4_, 0.2g KH_2_PO_4_, 0.20 g MgSO_4_.7H_2_O, 0.253 mg sodium molybdate (Na_2_MoO_4_), 0.13 g calcium sulphate (CaSO_4_) and 20 g agar.

Liquid media were of the same composition, without agar addition. The final pH of the media was set to 7.0 ± 0.1. For iron-replete media, 29 mg of iron(III) chloride (FeCl_3_) was also added.

Table S1 –Nutrient solutions used to balance the macronutrients.

|  | **Nutrient concentration (mol L^-1^)** | | | |
| --- | --- | --- | --- | --- |
| Solution | Ca(NO_3_)_2_ | KH_2_PO_4_ | K_2_HPO_4_ | MgSO_4_ |
| A | 0.57 | - | - | - |
| B | - | 0.16 | 0.04 | - |
| C | - | - | - | 0.04 |
| D | - | 0.22 | 0.10 | - |


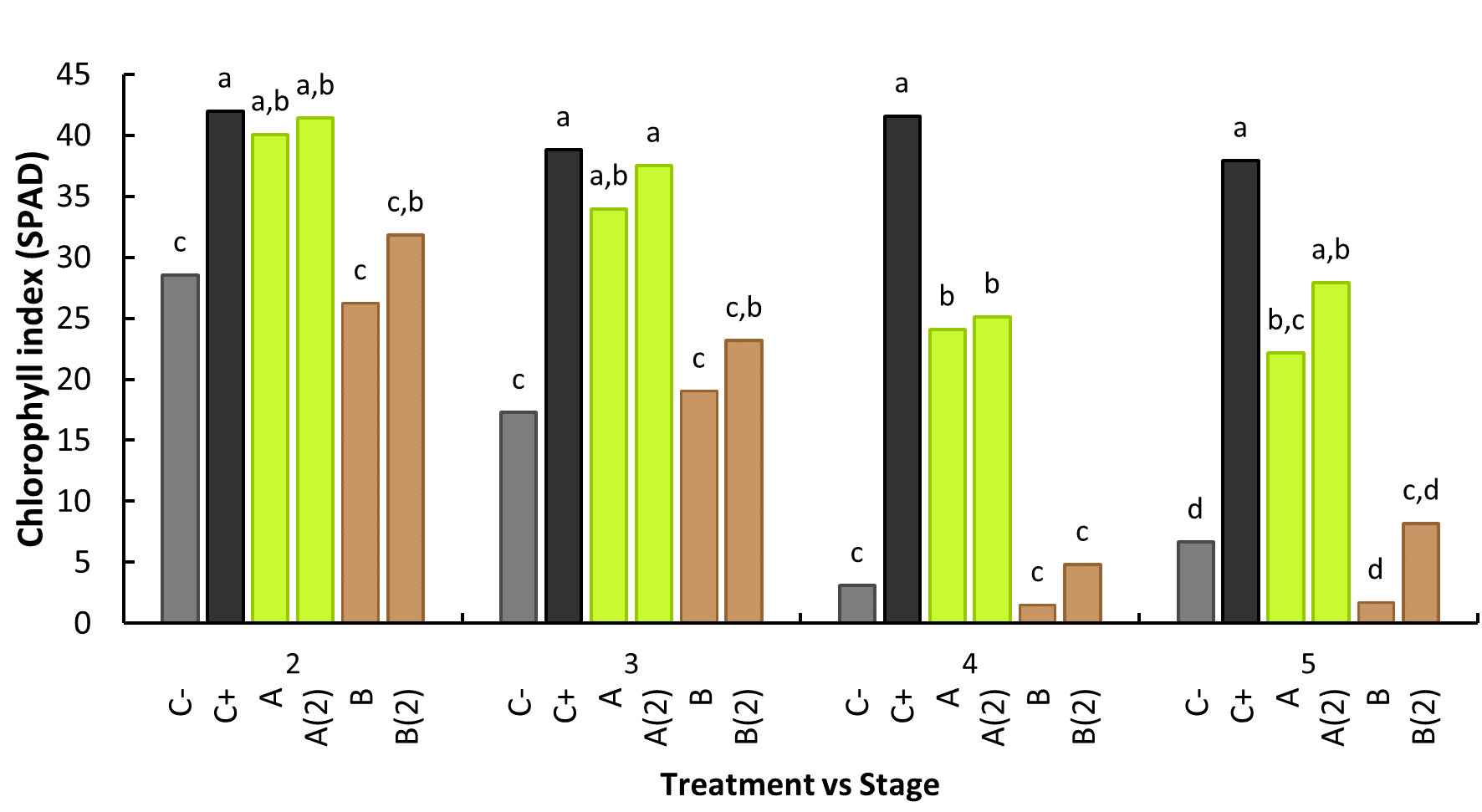


Figure S1. Intra-leaf stage (from 2^nd^ to 5^th^ stage) treatment comparison of average SPAD read at DAT 21. Different letters denote a significant difference of SPAD levels within each leaf stage within 95% confidence interval, as shown by Tuckey HSD test. C-: no iron treatment (negative control); C+: EDDHA (positive control); A: A. vinelandii ISS; B: B. subtilis ISS. Treatments with (2) represent plants with a second application performed 15th day after the first treatment (n=5).


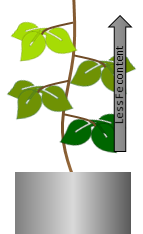


Figure S2. General model of leaf SPAD and Fe content progression on Azotobacter vinelandii ISS treated plants.

1. Bibliography

HiMedia Laboratories, 2015. Burks Medium - M707. Mumbai, India. http://himedialabs.com/TD/M707.pdf. Accessed 15 May 2015

Newton, J.W., Wilson, P.W., Burris, R.H., 1953. Direct demonstration of ammonia as an intermediate in nitrogen fixation by *Azotobacter*. J. Biol. Chem. 204, 445–451.
